# Synthesis and Antioxidant Activity of *N*-Benzyl-2-[4-(aryl)-1*H*-1,2,3-triazol-1-yl]ethan-1-imine Oxides

**DOI:** 10.3390/ijms25115908

**Published:** 2024-05-29

**Authors:** Dimitra Hadjipavlou-Litina, Iwona E. Głowacka, José Marco-Contelles, Dorota G. Piotrowska

**Affiliations:** 1 Laboratory of Pharmaceutical Chemistry, School of Pharmacy, Faculty of Health Sciences, Aristotle University of Thessaloniki, 54124 Thessaloniki, Greece; 2 Bioorganic Chemistry Laboratory, Faculty of Pharmacy, Medical University of Lodz, Muszyńskiego 1, 90-151 Lodz, Poland; iwona.glowacka@umed.lodz.pl; 3 Laboratory of Medicinal Chemistry, Institute of General Organic Chemistry (CSIC), Juan de la Cierva 3, 28006 Madrid, Spain; jlmarco@iqog.csic.es; 4 Centre for Biomedical Network Research on Rare Diseases (CIBERER), CIBER, ISCIII, 46010 Madrid, Spain

**Keywords:** anti-inflammatory activities, antioxidant activities, lipoxygenase inhibitors, lipid peroxidation, nitrones, synthesis

## Abstract

The synthesis, antioxidant capacity, and anti-inflammatory activity of four novel *N*-benzyl-2-[4-(aryl)-1*H*-1,2,3-triazol-1-yl]ethan-1-imine oxides **10a**–**d** are reported herein. The nitrones **10a**–**d** were tested for their antioxidant properties and their ability to inhibit soybean lipoxygenase (LOX). Four diverse antioxidant tests were used for in vitro antioxidant assays, namely, interaction with the stable free radical DPPH (1,1-diphenyl-2-picrylhydrazyl radical) as well as with the water-soluble azo compound AAPH (2,2′-azobis(2-amidinopropane) dihydrochloride), competition with DMSO for hydroxyl radicals, and the scavenging of cationic radical ABTS^•+^ (2,2′-azino-bis(3-ethylbenzothiazoline-6-sulfonate) radical cation). Nitrones **10b**, **10c,** and **10d**, having the 4-fluorophenyl, 2,4-difluorophenyl, and 4-fluoro-3-methylphenyl motif, respectively, exhibited high interaction with DPPH (64.5–81% after 20 min; 79–96% after 60 min), whereas nitrone **10a** with unfunctionalized phenyl group showed the lowest inhibitory potency (57% after 20 min, 78% after 60 min). Nitrones **10a** and **10d**, decorated with phenyl and 4-fluoro-3-methylphenyl motif, respectively, appeared the most potent inhibitors of lipid peroxidation. The results obtained from radical cation ABTS^•+^ were not significant, since all tested compounds **10a**–**d** showed negligible activity (8–46%), much lower than Trolox (91%). Nitrone **10c**, bearing the 2,4-difluorophenyl motif, was found to be the most potent LOX inhibitor (IC_50_ = 10 μM).

## 1. Introduction

Oxidative stress (OS) is a state of imbalance between the production and accumulation of free oxygen radicals in cells and tissues and the ability of the antioxidant system to remove them [1]. Antioxidants are chemical compounds that effectively neutralize the formation of free radicals. Their task is primarily to protect the body against free radicals, the excess of which may increase the risk of inflammation, arteriosclerosis, heart attacks, stroke, as well as neurodegenerative diseases (e.g., Parkinson’s and Alzheimer’s), among others [2,3,4]. The action of antioxidants is multidirectional, however, and the individual antioxidants differ in their mode of action. They may act either by multiple mechanisms or by a predominant mechanism [5,6,7]. Moreover, the biological importance of antioxidants is closely related to understanding the mechanisms of their action, which in turn determines the possibility of their practical use.

**Figure 1:**
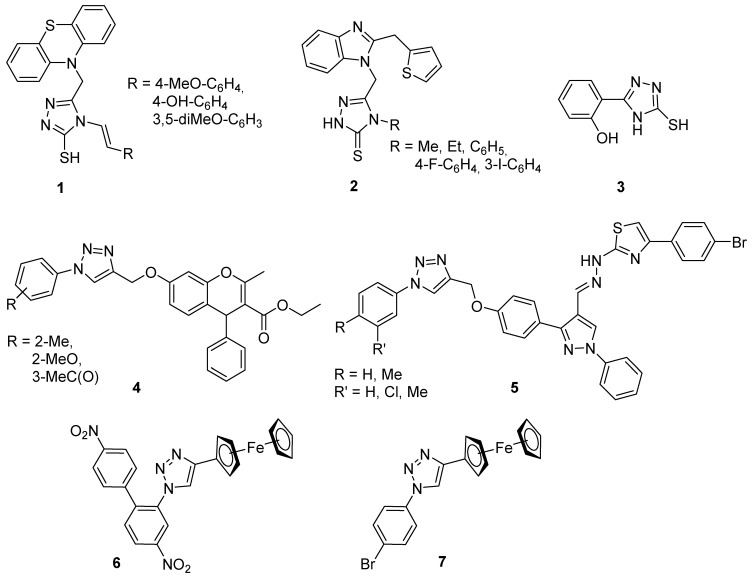
Structures of selected 1,2,4- and 1,2,3-triazole-containing compounds **1**–**7** with antioxidant activity.

A vast number of natural and synthetic compounds have been tested for their antioxidant properties over decades. Among them, nitrogen-containing five-membered heterocyclic compounds, including 1,2,3- and 1,2,4-triazoles [8,9], are of special importance due to the relatively simple method for their preparation and the possibility to modify their structure by incorporation into the more complex molecules (Figure 1). For example, the hybrids of functionalized 1,2,4-triazoles and phenothiazone **1** (Figure 1) appeared to be good antioxidants [10]. Furthermore, 1,2,4-triazoles **2** (Figure 1) conjugated with two other heterocyclic systems, namely benzimidazole and thiophene, have been recognized to exhibit very good (2,2′-azino-bis(3-ethylbenzothiazoline-6-sulfonic acid)) (ABTS) scavenging activity [11]. Compound **3** (Figure 1) showed high 1, 1-diphenyl-2-picrylhydrazyl radical (DPPH) scavenging activity with the percent inhibition of 93.751 ± 0.47 at a concentration of 100 µg/mL, and with IC_50_ value 7.12 ± 2.32 µg/mL was found to be more active than the standard antioxidant BHA (butylated hydroxyanisole) [12]. 4*H*-Chromene-containing 1,2,3-triazoles **4** (Figure 1) showed good antioxidant activity by DPPH and hydrogen peroxide radical scavenging methods [13]. Moreover, 1,2,3-triazoles containing both pyrazole and thiazole moieties **5** (Figure 1) have also been recognized as potent DPPH scavenging agents [14]. Ferrocene-1*H*-1,2,3-triazole hybrids **6** and **7** (Figure 1) exhibit antioxidant effects on mitochondrial free radicals and anti-inflammatory effects on rat mesangial cells (RMCs) [15].

**Figure 2:**
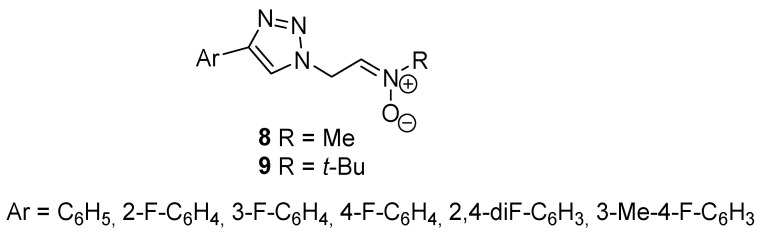
Structures of the compounds **8** and **9** encountered in the previous work [16].

Recently, we have investigated the antioxidant capacity of *N*-[2-(4-aryl-1*H*-1,2,3-triazol-1-yl)ethylidene]methanamine oxides **8** and *N*-[2-(4-aryl-1*H*-1,2,3-triazol-1-yl)ethylidene]-2-methylpropan-2-amine oxides **9** (Figure 2) [16]. Among all the tested nitrones, *N*-*tert*-butyl derivatives **9** (Figure 2) having the 4-fluorophenyl, 2,4-difluorophenyl, and 4-fluoro-3-methylphenyl substituents at C4 in 1,2,3-triazole moiety appeared the most potent hydroxyl radical scavengers (~100%), more potent than Trolox (88%), used as a reference compound. Moreover, *N*-{2-[4-(4-fluoro-3-methylphenyl)-1*H*-1,2,3-triazol-1-yl]ethylidene}-2-methylpropan-2-amine oxide **9** (R = *t*-Bu, Ar = 3-Me-4-F-C_6_H_3_) (Figure 2) was identified as the most balanced and potent antioxidant agent, since it was an extremely efficient and potent hydroxyl radical scavenger, the most potent 5-lipoxygenase (LOX) inhibitor, and one of the most potent lipid peroxidation inhibitors (LPis) and 2,2′-azino-bis(3-ethylbenzothiazoline-6-sulfonate) radical cation (ABTS^•+^) scavenger of the whole series of the tested nitrones.

**Figure 3:**
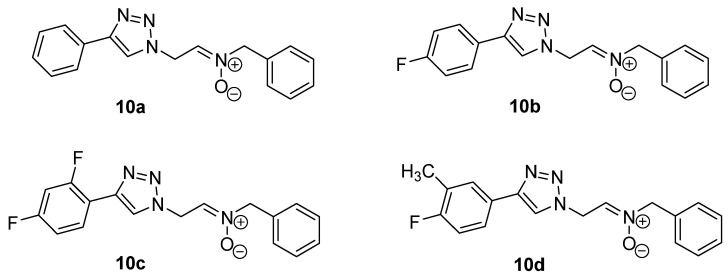
Structures of the nitrones **10a**–**d** reported in the present work.

In continuation to our studies to identify new nitrones for the therapy for pathological inflammation and oxidative stress (OS), the nitrones **10a**–**d** (Figure 3), *N*-benzyl analogs of the previously reported compounds **8** and **9** [16] (Figure 2), have been synthesized with the intention of testing their antioxidant potency. The newly prepared series of compounds contains unfunctionalized nitrone **10a** as well as derivatives substituted at the C4 of 1,2,3-triazole moiety with 4-fluorophenyl (**10b**), 2,4-difluorophenyl (**10c**), and 4-fluoro-3-methylphenyl (**10d**), which were selected from the previously synthesized nitrones of series **8** and **9** [16] based on their observed antioxidant activity.

## 2. Results and Discussion

### 2.1. Chemistry

**Scheme 1:**
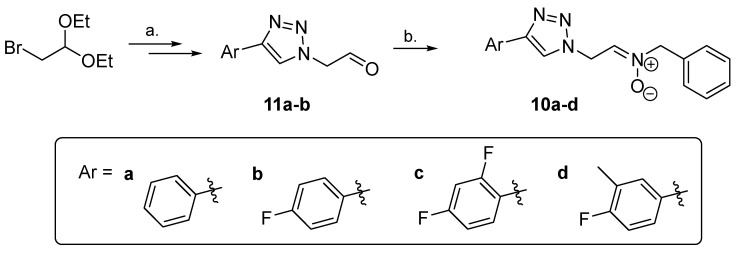
Synthesis of nitrones **10a**–**d**. *Reagents and conditions*: a. details for preparation of aldehydes **11a**–**d** given in [16]; b. BnNHOH × HCl, CH_3_CO_2_Na, rt, 15 min.

Nitrones **10a**–**d** were prepared following the reactions shown in Scheme 1 as previously described [16], by reacting aldehydes **11a**–**b** with *N*-benzylhydroxylamine. The progress of the reaction was monitored by TLC and the full conversion of the aldehydes **11a**–**b** into respective nitrones **10** was achieved within 15 min, at room temperature (rt). All final products were purified by crystallization and their structure and purities were established by ^1^H, ^13^C, and ^19^F NMR (Supplementary Materials, Figures S1–S11), and IR techniques and by elemental analysis (Section 3). In particular, nitrones **10a**–**d** were isolated as pure *Z*-stereoisomers at the double bond (CH=N), as determined and confirmed by the presence of the single sets of the diagnostic signals of the respective protons CH=N (*δ* = 7.12–7.15 ppm) and C*H*_2_Ph (*δ* = 4.97–5.00 ppm) in the ^1^H NMR spectra of **10a**–**d**. Based on a comparison of the literature data for other acyclic nitrones [17], Z-configuration was assigned for (*Z*)-**10a**–**d**; however, the corresponding signals for the *E*-isomeric nitrones necessary to provide full correlation were not observed.

### 2.2. In Vitro Antioxidant and Anti-Inflammatory Activity

Herein, we have investigated in vitro the antioxidant evaluation of nitrones **10a**–**d** with regard to their antioxidant ability as well as to their ability to inhibit soybean LOX on several diverse antioxidant tests and in comparison to nordihydroguaiaretic acid (NDGA) and Trolox as standards. All aerobic organisms produce free radicals that can attack and damage lipids and DNA, inducing neurodegenerative diseases, cancer, and stroke. Since OS and inflammation present a complex character, we decided to evaluate the in vitro antioxidant activity of the synthesized molecules using four different antioxidant assays:

(a) Interaction with the stable free radical DPPH;
(b) Interaction with the water-soluble azo compound 2,2′-azobis(2-amidinopropane) dihydrochloride (AAPH);
(c) Competition with DMSO for hydroxyl radicals;
(d) The scavenging of cationic radical ABTS^•+^.

All are spectrophotometric measurements which are simple, rapid, and convenient.

DPPH is a stable free radical, advantageous for testing compounds in an ethanolic solution, which in its oxidized form presents a maximum absorbance at about 517 nm. The DPPH method is independent of the molecule’s polarity. The reducing activity (RA) of the examined compounds with the stable free radical DPPH is given in Table 1. This interaction shows their radical scavenging ability in an iron-free system. Nitrones **10b**, **10c,** and **10d** highly interact with DPPH (64.5–81%) after 20 min, whereas **10a** presents a lower value. In general, the insertion of a substituent increases the reducing activity (RA). Thus, starting from **10a**, all the other compounds in which one or two fluorine atoms or a fluorine atom and a methyl group are present, exhibit higher activities. It seems that an acceptor, such as a fluorine atom, with small molar refractivity (MR) in the para position offers antioxidant ability. The presence of a second fluorine atom as a substituent, **10c** does not influence the interaction values, whereas a methyl group in *meta* position acting as a donor lowers activity (**10b** > **10d**). RA is not influenced by lipophilicity within this nitrones group. The interaction values are increased for all after 60 min (78–81%), showing time dependency. For the sake of comparison, NDGA was used as a standard reference compound.

In our studies, the water-soluble azo AAPH was used as a thermal free radical initiator to induce the oxidative changes of linoleic acid to conjugated diene hydroperoxide. All nitrones except for **10c** presented inhibition values (75–87%) lower than the common standard Trolox (93%) (Table 1). The compounds **10a** and **10d** are the most potent. Lipophilicity seems to play a significant role related to a positive result since the inserted methyl group in compound **10b** (75%) increases the lipophilicity of compound **10d** driving it to higher inhibition (87%). Nitrone **10c** having two fluorine atoms and a clogP value of 2.24 exhibits the lowest anti-lipid peroxidation activity.

Hydroxyl (^•^OH) free radical is counted as the most toxic. As a result, it reacts with important biological molecules such as DNA, lipids, or carbohydrates. We found it interesting to test the scavenging activity of the compounds in competition with DMSO. As shown in Table 1, all the compounds do not exhibit any activity compared to the standard compound Trolox.

In the ABTS^•+^ decolorization assay, the tested nitrones showed very low activity, except for nitrone **10b** which is a mono-substituted fluor derivative. The compounds **10c** and **10d** exhibit equipotent results, lower activity than **10b** (almost the half), and higher lipophilicity values.

We evaluated the synthesized nitrones for their ability to inhibit soybean LOX by the UV absorbance-based enzyme protocol, as shown in Table 1 [18]. The appropriate stimulation of neutrophils cleaves arachidonic acid (AA) from membrane phospholipids, producing leukotrienes through lipoxygenase. Leukotriene B4 (LTB4) is a potent mediator of inflammation, considered to be important in the pathogenesis of neutrophil-mediated inflammatory diseases with a marked relation to the severity of cardiovascular diseases, stroke, and cancer [19]. The enzyme lipoxygenase catalyzes the first two steps in the metabolism of AA, which is cleaved from membrane phospholipids to leukotrienes (LTB4). LTB4 generation is important in the pathogenesis of neutrophil-mediated inflammatory diseases. NDGA, a known inhibitor of soybean LOX, has been used as a reference compound with IC_50_ 0.45 µM. A perusal of the IC_50′_s inhibition values (Table 1) shows that the most potent inhibitors are the compounds **10c** (IC_50_ 10 μM), **10b** (IC_50_ 62.5 μM), and **10a** (IC_50_ 85 μM). Compound **10d** presents a lower activity of 45% at 100 μM. The structural moiety that significantly influences the inhibition in compounds **10b** and **10c** is the fluorine atom. In both compounds, this electronegative substituent is present. The most potent nitrone **10c** possesses two fluorine atoms whereas **10b** has one. The loss of the second fluorine atom (**10b**) lowers the activity as well as the absence of nitrone **10a**. Substituents with low bulk, such as fluorine, and lipophilic contribution as π values increase the inhibitory activity. The strong inhibition of **10c** could be therapeutically useful in stroke or neurodegeneration in combination with the high RA (%). It is worth mentioning that the high efficacy of the fluorinated derivatives of PBN (α-phenyl-N-tert-butyl nitrone), namely 4-F-PBN and 4-CF_3_-PBN, for spin-trapping experiments when compared to PBN has been recently described by Durand and co-workers [20].

## 3. Materials and Methods

### 3.1. Chemistry

General information—The ^1^H, ^13^C, and NMR spectra were taken in CDCl_3_ on the Bruker Avance III spectrometers (600 MHz, Bruker Instruments, Karlsruhe, Germany) with TMS as the internal standard at 600 and 151 MHz, respectively. The ^19^F NMR spectra were recorded in CDCl_3_ on the Bruker AvanceNEO (Bruker Instruments, Karlsruhe, Germany) at 565 MHz. The IR spectra were measured on an Infinity MI-60 FT-IR spectrometer (Bruker Optik GmbH, Ettlingen, Germany). The melting points were determined on a Boetius apparatus and are uncorrected. The elemental analyses were performed by the Microanalytical Laboratory of this Faculty on the Perkin-Elmer PE 2400 CHNS analyzer (Perkin Elmer Corp., Norwalk, CT, USA). The following adsorbents were used: column chromatography, Merck silica gel 60 (70–230 mesh); analytical TLC, Merck TLC plastic sheets silica gel 60 F_254_ (Merck KGaA, Darmstadt, Germany).

The ^1^H-, ^13^C-, and ^19^F-NMR spectra of all the newly synthesized compounds are provided in Supplementary Materials.

### 3.2. General Procedure for the Preparation of Nitrones ***10a**–**d***

The respective aldehydes **11a**–**d**, obtained directly from corresponding diethyl acetal according to the previously described procedure [13], were dissolved in ethanol (2 mL), and CH_3_CO_2_Na (1.3 mmol) was added followed by *N*-benzylhydoxylamine hydrochloride (1.1 mmol). The reaction mixture was stirred until the disappearance of the starting aldehyde was noticed on TLC. After that, 10% NaHCO_3_ was added (5 mL) and the product was extracted with methylene chloride (3 × 5 mL). The organic extracts were combined, dried (MgSO_4_), concentrated, and crystallized from diethyl ether to give the pure nitrone **10a**–**d**.

#### 3.2.1. *N*-Benzyl-2-(4-phenyl-1*H*-1,2,3-triazol-1-yl)ethan-1-imine Oxide (**10a**)

Yield 79%; white amorphous solid; m.p. 134–136 °C (recrystallized from diethyl ether); IR (KBr, cm^–1^) ν_max_: 3398, 3347, 3130, 3033, 2948, 764, and 696. ^1^H NMR (600 MHz, CDCl_3_): δ = 7.95 (s, 1H, *H*C5′), 7.84 (d, *J* = 7.3 Hz, 2H, H_aromat._), 7.46–7.43 (m, 7H, H_aromat._), 7.37 (t, *J* = 7.3 Hz, 1H, H_aromat._), 7.14 (t, *J* = 5.4 Hz, 1H, =C*H*CH_2_), 5.36 (d, *J* = 5.4 Hz, 2H, =CHC*H*_2_), and 4.97 (s, 2H, C*H*_2_Ph); ^13^C NMR (151 MHz, CDCl_3_): δ = 148.12, 131.66, 130.93, 130.29, 129.56, 129.21, 128.86, 128.31, 125.78, 120.95, 69.80, and 45.78. Anal. calcd. for C_17_H_16_N_4_O × 0.5H_2_O: C, 67.76; H, 5.69; N, 18.59. Found: C, 67.55; H, 5.60; N, 18.34.

#### 3.2.2. *N*-Benzyl-2-[4-(4-fluorophenyl)-1*H*-1,2,3-triazol-1-yl]ethan-1-imine Oxide (**10b**)

Yield 93%; white amorphous solid; m.p. 129–131 °C (recrystallized from diethyl ether); IR (KBr, cm^–1^) ν_max_: 3321, 3094, 3074, 2956, and 835. ^1^H NMR (600 MHz, CDCl_3_): δ = 7.95 (s, 1H, *H*C5′), 7.82–7.79 (m, 2H, H_aromat._), 7.45–7.43 (m, 5H, H_aromat._), 7.15–7.13 (m, 3H, 2 × H_aromat._, =C*H*CH_2_), 5.38 (d, *J* = 5.5 Hz, 2H, =CHC*H*_2_), and 4.99 (s, 2H, C*H*_2_Ph); ^13^C NMR (151 MHz, CDCl_3_): δ = 162.78 (d, *J* = 246.6 Hz), 147.23, 131.63, 130.69, 129.56, 129.21, 127.52 (d, *J* = 7.8 Hz), 126.52 (d, *J* = 3.5 Hz), 120.75, 115.86 (d, *J* = 21.8 Hz), 69.85, and 45.69; ^19^F NMR (565 MHz, CDCl_3_): δ = −113.30–−113.35 (m). Anal. calcd. for C_17_H_15_FN_4_O × 0.5H_2_O: C, 63.94; H, 5.05; N, 17.54. Found: C, 63.80; H, 4.91; N, 17.44.

#### 3.2.3. *N*-Benzyl-2-[4-(2,4-difluorophenyl)-1*H*-1,2,3-triazol-1-yl]ethan-1-imine Oxide (**10c**)

Yield 78%; white amorphous solid; m.p. 112–113 °C (recrystallized from diethyl ether); IR (KBr, cm^–1^) ν_max_: 3321, 3158, 3030, 2939, 2904, and 736. ^1^H NMR (600 MHz, CDCl_3_): δ = 8.27 (dt, *J* = 8.4 Hz, *J* = 6.4 Hz, 1H), 8.06 (d, *J* = 3.6 Hz, 1H), 7.44 (s, 5H), 7.12 (t, *J* = 5.4 Hz, 1H), 7.05–7.00 (m, 1H), 6.93 (ddd, *J* = 10.7 Hz, *J* = 8.4 Hz, *J* = 2.5 Hz, 1H), 5.41 (d, *J* = 5.4 Hz, 2H), and 5.00 (s, 2H, C*H*_2_Ph); ^13^C NMR (151 MHz, CDCl_3_): δ = 162.64 (dd, *J* = 250.4 Hz, *J* = 11.9 Hz), 159.28 (dd, *J* = 250.7 Hz, *J* = 11.9 Hz), 140.89 (d, *J* = 2.0 Hz), 131.62, 130.79, 129.59, 129.22, 128.80 (dd, *J* = 9.5 Hz, *J* = 4.7 Hz), 123.46 (d, *J* = 12.1 Hz), 112.04 (dd, *J* = 21.7 Hz, *J* = 4.1 Hz), 104.16 (dd, *J* = 25.9 Hz, *J* = 25.2 Hz), 69.79, and 46.03; ^19^F NMR (565 MHz, CDCl_3_): δ = −109.93–−110.01 (m), −110.70–−110.76 (m). Anal. calcd. for C_17_H_14_F_2_N_4_O × 2H_2_O: C, 56.04; H, 4.98; N, 15.38. Found: C, 56.18; H, 5.01; N, 15.41.

#### 3.2.4. *N*-Benzyl-2-[4-(4-fluoro-3-methylphenyl)-1*H*-1,2,3-triazol-1-yl]ethan-1-imine Oxide (**10d**)

Yield 85%; white amorphous solid; m.p. 131–133 °C (recrystallized from diethyl ether); IR (KBr, cm^–1^) ν_max_: 3320, 3136, 3036, 2935, 820, and 736. ^1^H NMR (600 MHz, CDCl_3_): δ = 7.93 (s, 1H), 7.68 (dd, *J* = 7.3 Hz, *J* = 1.6 Hz, 1H), 7.60–7.57 (m, 1H), 7.46–7.41 (m, 5H_._), 7.13 (t, *J* = 5.5 Hz, 1H), 7.07 (t, *J* = 8.9 Hz, 1H), 5.37 (d, *J* = 5.5 Hz, 2H), 4.99 (s, 2H, C*H*_2_Ph), and 2.35 (d, *J* = 1.7 Hz, 3H); ^13^C NMR (151 MHz, CDCl_3_): δ = 161.35 (d, *J* = 246.6 Hz), 147.43, 131.61, 130.82, 129.59, 129.22, 128.94 (d, *J* = 5.4 Hz), 126.12 (d, *J* = 4.1 Hz), 125.43 (d, *J* = 18.4 Hz), 124.44 (d, *J* = 8.0 Hz), 120.68, 115.46 (d, *J* = 22.7 Hz), 69.83, 45.70, and 14.56 (d, *J* = 3.3 Hz); ^19^F NMR (565 MHz, CDCl_3_): δ = −117.65–−117.89 (br m). Anal. calcd. for C_18_H_17_FN_4_O × H_2_O: C, 63.15; H, 5.59; N, 16.36. Found: C, 63.18; H, 5.46; N, 16.32.

### 3.3. In Vitro Assays

General biological assays: NDGA, Trolox, AAPH, and DPPH soybean LOX linoleic acid sodium salt were purchased from the Aldrich Chemical Co., Milwaukee, WI, USA. The phosphate buffer (0.1 M and pH 7.4) was prepared by mixing an aqueous KH_2_PO_4_ solution (50 mL, 0.2 M), and an aqueous NaOH solution (78 mL, 0.1 M); the pH (7.4) was adjusted by adding a solution of KH_2_PO_4_ or NaOH. For the in vitro tests, a Lambda 20 (Perkin–Elmer-PharmaSpec 1700, Perkin-Elmer Corporation Ltd., Lan Beaconsfield, Bucks, UK) UV–Vis double beam spectrophotometer was used. Each in vitro experiment was performed at least in triplicate and the standard deviation of absorbance was less than 10% of the mean.

#### 3.3.1. Determination of the RA of the Stable Radical DPPH [21]

To an ethanolic solution of DPPH (0.05 mM) in absolute ethanol, 10 µL from a stock solution (10 mM) in the DMSO of the compounds was added. The mixture was shaken vigorously and allowed to stand for 20 min or 60 min; absorbance at 517 nm was determined spectrophotometrically and the percentage of activity was calculated. All tests were undertaken on three or four replicates and the results were averaged (Table 1). NDGA was used as a reference compound.

#### 3.3.2. Soybean LOX Inhibition Study In Vitro [22]

The tested compounds dissolved in DMSO were incubated at rt with sodium linoleate (0.1 mL) and 0.2 mL of the enzyme solution (1/9 × 10^−4^ *w*/*v* in saline) in buffer Tris pH 9. The conversion of sodium linoleate to 13-hydroperoxylinoleic acid at 234 nm was recorded and compared with NDGA, the appropriate standard inhibitor (Table 1).

#### 3.3.3. Inhibition of Linoleic Acid Lipid Peroxidation [16]

Ten microliters of the 16 mM linoleic acid sodium salt solution were added to the UV cuvette containing 0.93 mL of the 0.05 M phosphate buffer, pH 7.4 prethermostated at 37 °C. The oxidation reaction was initiated at 37 °C under air by the addition of 50 μL of the 40 mM AAPH solution. Oxidation was carried out in the presence of 10 μL of the compounds’ stock 10 mM solution in DMSO, in the assay. Lipid oxidation was measured in the presence of the same level of DMSO. The rate of oxidation at 37 °C was recorded as the absorption values at 234 nm and compared to Trolox (Table 1).

#### 3.3.4. Competition of the Tested Compounds with DMSO for Hydroxyl Radicals [23]

The hydroxyl radicals were produced by the Fe^3+/^ascorbic acid system and detected by the determination of the formaldehyde produced from the oxidation of DMSO. EDTA (0.1 mM), Fe^3+^ (167 μM), DMSO (33 mM) in phosphate buffer (50 mM, pH 7.4), the tested compounds (100 µM) and ascorbic acid (10 mM) were inserted, mixed, and incubated in test tubes at 37 °C for 30 min. The reaction was stopped by CCl_3_COOH (17% *w*/*v*) and the % scavenging activity of the nitrones for hydroxyl radicals was recorded. Trolox was used as a positive control (Table 1).

#### 3.3.5. ABTS^•+^–Decolorization Assay in Ethanolic Solution for Antioxidant Activity [23]

ABTS^•+^ was produced according to the described procedure as follows. An ABTS stock solution in water (7 mM) was mixed with potassium persulfate (2.45 mM) and left in a dark at room temperature for 12–16 h before use. A total of 10 µL of the investigated compounds were added to ethanol together with the cationic radical. The results were taken at 734 nm, after 1 min of the mixing procedure. Trolox was used as a positive standard (Table 1).

#### 3.3.6. Estimation of Lipophilicity as Clog P

Bioloom of Biobyte Corp was used for the theoretical calculation of lipophilicity as Clog *P* values (BioByte Home Page. Available online: http://www.biobyte.com, accessed on 1 April 2024). We followed this procedure because lipophilicity is an important physicochemical property related to the biological activity and Absorbance–Distribution–Metabolism–Toxicity (ADMET) properties.

## 4. Conclusions

The synthesized nitrones **10a**–**d** were evaluated for their antioxidant activity using different in vitro techniques. Their anti-inflammatory activity was also tested. Thus, the inhibition of soybean LOX was developed as an indication of their anti-inflammatory effect. The in vitro results revealed that compound **10c** is the most promising LOX inhibitor (IC_50_ = 10 μM) combining a significant anti-lipid peroxidation activity (79%). It seems that **10c** could be a promising lead compound to confront inflammatory diseases where OS has been identified to be crucial. Further research is now in progress in our laboratory and will be reported elsewhere.

## Figures and Tables

**Table 1:** Antioxidant activity of nitrones **10a**–**d** ^a^, and standards Trolox and NDGA.

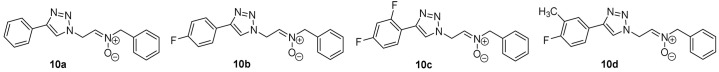						
**Nitrones/** **Standards**	**Clog *P* ^b^**	**ILP** **(%)**	**RA (%)** **20 min/60 min**	**LOX Inhibition** **(% or IC_50_ [µM])**	**Scavenging Activity for HO^•^ (%) ^a^**	**ABTS^•+^** **(%) ^a^**
**10a**	1.91	81	57/78	85 μM	no	8
**10b**	2.09	75	81/81	62.5 μM	no	46
**10c**	2.24	32	79/79	10 μM	no	23
**10d**	2.59	87	64.4/79.5	45%	no	23
NDGA	3.92	nt	88/96	0.45 μM	nd	-
Trolox	3.09	93	nd	nd	73	91

^a^ nitrones tested at 100 µM. Values are the means of three or four different determinations. no = no activity under the experimental conditions. Means within each column differ significantly (*p* < 0.05). ^b^ BioByte Corporation, C-QSAR database, 201 W Fourth Str., Suite # 204, Claremont CA 91711-4707, USA. nd, not determined.

## Data Availability

Data are contained within the article.

## Supplementary Materials

The following supporting information can be downloaded at: https://www.mdpi.com/article/10.3390/ijms25115908/s1.

## Abbreviations

AAPH, 2,2′-Azobis(2-amidinopropane) dihydrochloride; ABTS: 2,2′-azino-bis(3-ethylbenzothiazoline-6-sulfonic acid); ABTS^•+^, 2,2′-azino-bis(3-ethylbenzothiazoline-6-sulfonate) radical cation; ADMET, Absorbance–Distribution–Metabolism–Toxicity; BHA, butylated hydroxyanisole; DMSO, Dimethyl sulfoxide; DPPH, 1,1-diphenyl-2-picrylhydrazyl radical; EDTA, Ethylenediaminetetraacetic acid; ILP, inhibition of lipid peroxydation; LOX, lipoxygenase; LPis, lipid peroxidation inhibitors; LTB4, leukotriene B4; MR, molar refractivity; NDGA, Nordihydroguaretic acid; OS, oxidative stress; PBN, α-phenyl-N-tert-butyl nitrone; RA, reducing activity; RMCs, rat mesangial cells.

## References

[1] Lutskii M.A., Zemskov A.M., Razuvaeva V.V., Lushnikova Y.P., Karpova O.Y. (2018). Oxidative stress as an indicator of metabolic impairments in the pathogenesis of cerebral stroke. Neurosci. Behav. Physiol..

[2] Kontoghiorghes G.J., Kontoghiorghe C.N. (2019). Prospects for the introduction of targeted antioxidant drugs for the prevention and treatment of diseases related to free radical pathology. Expert Opin. Investig. Drugs..

[3] Ma A., Qi S., Chen H. (2008). Antioxidant therapy for prevention of inflammation, ischemic reperfusion injuries and allograft rejection. Cardiovasc. Hematol. Agents Med. Chem..

[4] Maxwell S.R. (1995). Prospects for the use of antioxidant therapies. Drugs.

[5] Halliwell B. (2024). Understanding mechanisms of antioxidant action in health and disease. Nat. Rev. Mol. Cell Biol..

[6] Hunyadi A. (2019). The mechanism(s) of action of antioxidants: From scavenging reactive oxygen/nitrogen species to redox signaling and the generation of bioactive secondary metabolites. Med. Res. Rev..

[7] Lu J.-M., Lin P.H., Yao Q., Chen C. (2010). Chemical and molecular mechanisms of antioxidants: Experimental approaches and model systems. J. Cell. Mol. Med..

[8] Mohammed J.H., Kadhim H.Y., Makki K.A., Ali B.A. (2021). Review on antioxidant evaluation ofn1,2,3-triazole derivatives synthesized by click chemistry. Ann. Rom. Soc. Cell Biol..

[9] Pachut-Stec A. (2022). Antioxidant activity of 1,2,4-triazole and its derivatives: A mini-review. Mini Rev. Med. Chem..

[10] Maddila S., Momin M., Gorle S., Palakondu L., Jonnalagadda S.B. (2015). Synthesis and antioxidant evaluation of novel phenothiazine linked substituted benzylideamino-1,2,4-triazole derivatives. J. Chil. Chem. Soc..

[11] Mentese E., Yilmaz F., Baltas N., Bekircan O., Kahveci B. (2015). Synthesis and antioxidant activities of some new triheterocyclic compounds containing benzimidazole, thiophene, and 1,2,4-triazole ring. J. Enzym. Inhib. Med. Chem..

[12] Cetin A., Gecibesler I.H. (2015). Evaluation as antioxidant agents of 1,2,4-triazole derivatives: Effects of essential functional groups. J. App. Pharm. Sci..

[13] Aitha S., Thumma V., Matta R., Ambala S., Jyothi K., Manda S., Pochampally J. (2023). Antioxidant activity of novel 4*H*-chromene tethered 1,2,3-triazole analogues: Synthesis and molecular docking studies. Results Chem..

[14] Matta R., Pochampally J., Dhoddi B.N., Bhookya S., Bitla S., Akkiraju A.G. (2023). Synthesis, antimicrobial and antioxidant activity of triazole, pyrazole containing thiazole derivatives and molecular docking studies. BMC Chem..

[15] Cheng C.-Y., Haque A., Hsieh M.F., Hassan S.I., Fazi M.S.H., Dege N., Khan M.S. (2020). 1,4-Disubstituted 1*H*-1,2,3-triazoles for renal diseases: Studies of viability, anti-inflammatory, and antioxidant activities. Int. J. Mol. Sci..

[16] Hadjipavlou-Latina D., Głowacka I.E., Marco-Contelles J., Piotrowska D.G. (2023). Synthesis and antioxidant properties of novel 1,2,3-triazole-containing nitrones. Antioxidants.

[17] Piotrowska D.G. (2006). N-Substituted C-diethoxyphosphorylated nitrones as useful synthons for the synthesis of α-aminophosphonates. Tetrahedron Lett..

[18] Hadjipavlou-Litina D., Bariamis S.E., Militsopoulou M., Athanassopoulos C.M., Papaioannou D. (2009). Trioxsalen derivatives with lipoxygenase inhibitory activity. J. Enzym. Inhib. Med. Chem..

[19] Muller K. (1994). 5-Lipoxygenase and 12-lipoxygenase: Attractive targets for the development of novel antipsoriatic drugs. Arch. Pharm..

[20] Deletraz A., Tuccio B., Roussel J., Combes M., Cohen-Solal C., Farbe P.-L., Trouillas P., Vignes M., Callizot N., Durand G. (2020). *Para*-Substituted α-Phenyl-*N*-tert-butyl Nitrones: Spin-Trapping, Redox and Neuroprotective Properties. ACS Omega.

[21] Koleva I.I., van Beek T.A., Linssen J.P.H., de Groot A., Evstatieva L.N. (2002). Screening of plant extracts for antioxidant activity: A comparative study on three testing methods. Phytochem. Anal..

[22] Liegois C., Lermusieau G., Colin S. (2000). Measuring antioxidant efficiency of wort, malt, and hops against the 2,2′-azobis(2-amidinopropane) dihydrochloride-induced oxidation of an aqueous dispersion of linoleic acid. J. Agric. Food Chem..

[23] Chamorro B., Diez-Iriepa D., Merás-Sáiz B., Chioua M., García-Vieira D., Iriepa I., Hadjipavlou-Litina D., López-Muñoz F., Martínez-Murillo R., González-Nieto D. (2020). Synthesis, antioxidant properties and neuroprotection of alpha-phenyl-tertbutylnitrone derived homobisnitrones in in vitro and in vivo ischemia models. Sci. Rep..

