# Supplementary material for: Synthesis and Antioxidant Activity of N-Benzyl-2-[4-(aryl)-1H-1,2,3-triazol-1-yl]ethan-1-imine Oxides
Source: Int J Mol Sci. 2024 May 29;25(11):5908. doi: 10.3390/ijms25115908 (PMC11173104; doi:10.3390/ijms25115908)
Supplement: Supplementary file 1 [file ijms-25-05908-s001.zip › ijms-3015148-supplementary.pdf]

Article

Synthesis and Antioxidant Activity of *N*-Benzyl-2-[4-(aryl)-1*H*-1,2,3-triazol-1-yl]ethan-1-imine Oxides

Dimitra Hadjipavlou-Litina [1,*], Iwona E. Głowacka [2], José Marco-Contelles [3,4] and Dorota G. Piotrowska [2,*]

[1] Laboratory of Pharmaceutical Chemistry, School of Pharmacy, Faculty of Health Sciences, Aristotle University of Thessaloniki, 54124 Thessaloniki, Greece
[2] Bioorganic Chemistry Laboratory, Faculty of Pharmacy, Medical University of Lodz, Muszyńskiego 1, 90-151 Lodz, Poland; iwona.glowacka@umed.lodz.pl
[3] Laboratory of Medicinal Chemistry, Institute of General Organic Chemistry (CSIC), Juan de la Cierva 3, 28006 Madrid, Spain; jlmarco@iqog.csic.es
[4] Centre for Biomedical Network Research on Rare Diseases (CIBERER), CIBER, ISCIII, 46010 Madrid, Spain
* Correspondence: hadjipav@pharm.auth.gr (D.H.-L.); dorota.piotrowska@umed.lodz.pl (D.G.P.)

Contents

NMR spectra for compounds **10a-10d**:

Figure S1: ^{1}H NMR Spectrum for **10a** in $CDCl_3$

Figure S2: ^{13}C NMR Spectrum for **10a** in $CDCl_3$

Figure S3: ^{1}H NMR Spectrum for **10b** in $CDCl_3$

Figure S4: ^{19}F NMR Spectrum for **10b** in $CDCl_3$

Figure S5: ^{13}C NMR Spectrum for **10b** in $CDCl_3$

Figure S6: ^{1}H NMR Spectrum for **10c** in $CDCl_3$

Figure S7: ^{19}F NMR Spectrum for **10c** in $CDCl_3$

Figure S8: ^{13}C NMR Spectrum for **10c** in $CDCl_3$

Figure S9: ^{1}H NMR Spectrum for **10d** in $CDCl_3$

Figure S10: ^{19}F NMR Spectrum for **10d** in $CDCl_3$

Figure S11: ^{13}C NMR Spectrum for **10d** in $CDCl_3$

Figure S1: ^{1}H NMR Spectrum for **10a** in $CDCl_3$

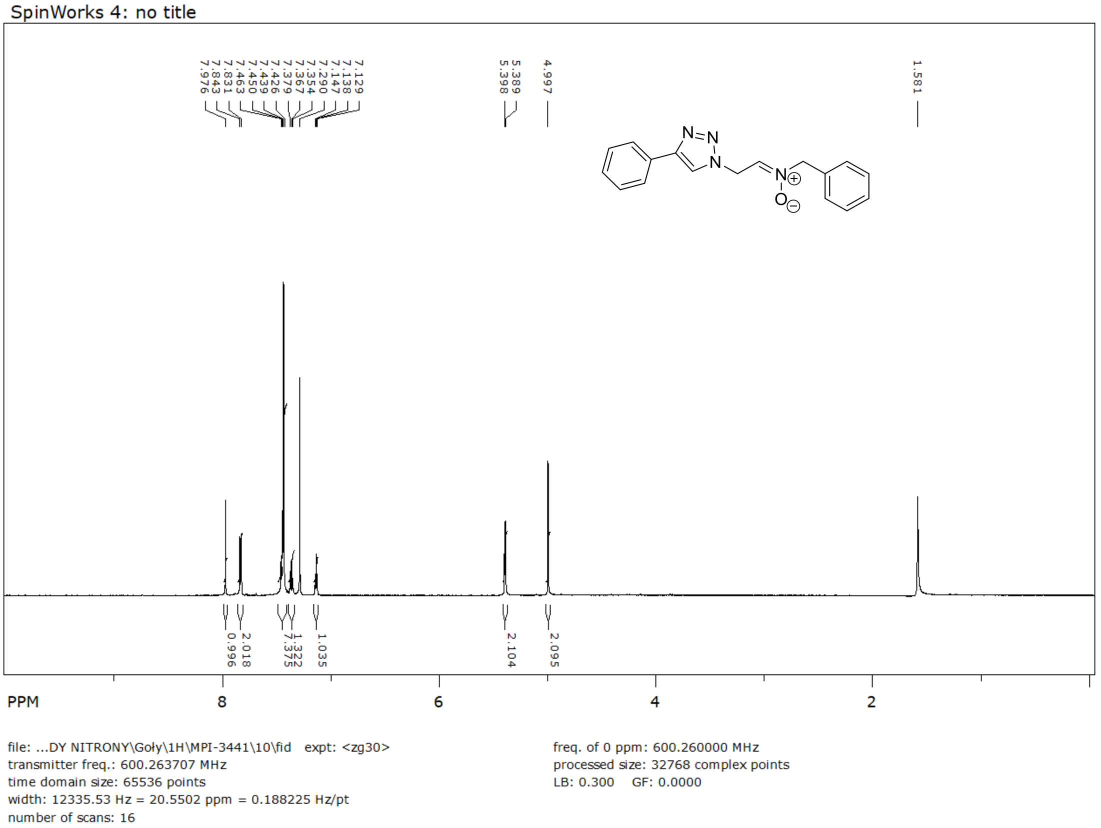

Figure S2: ^{13}C NMR Spectrum for **10a** in $CDCl_3$

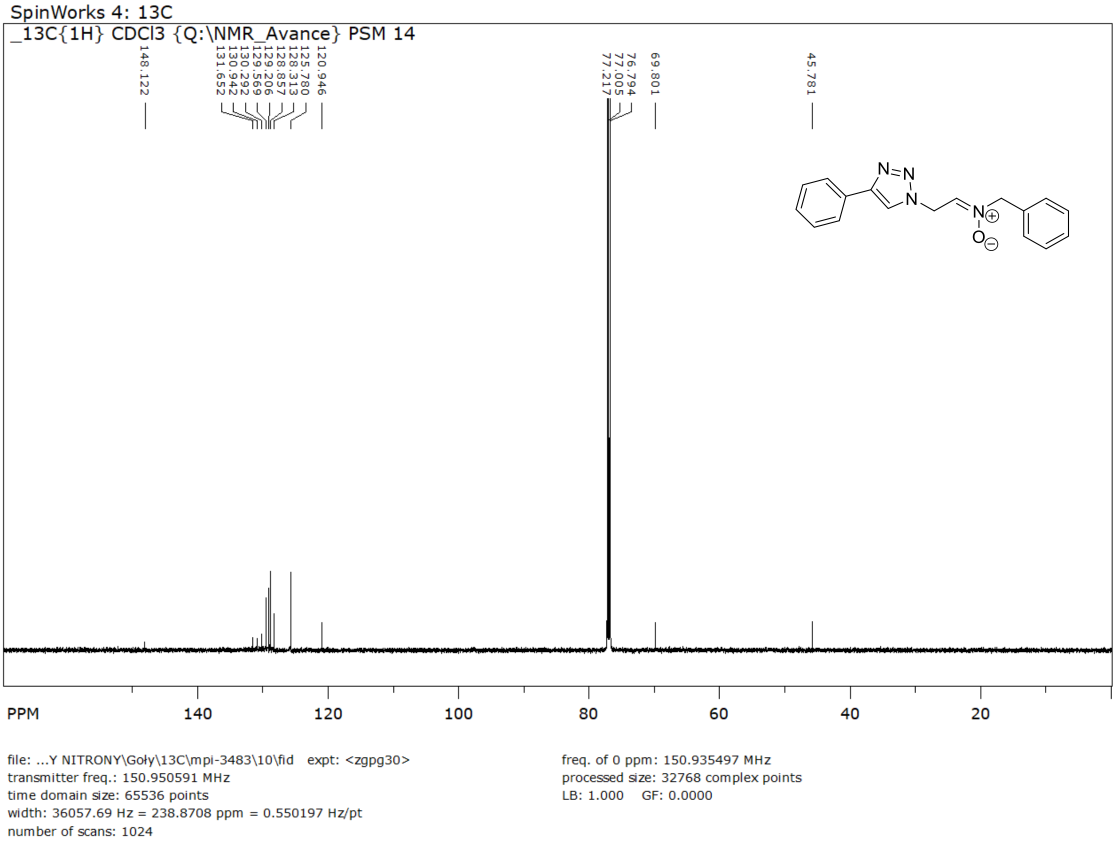

Figure S3: ^{1}H NMR Spectrum for **10b** in $CDCl_3$

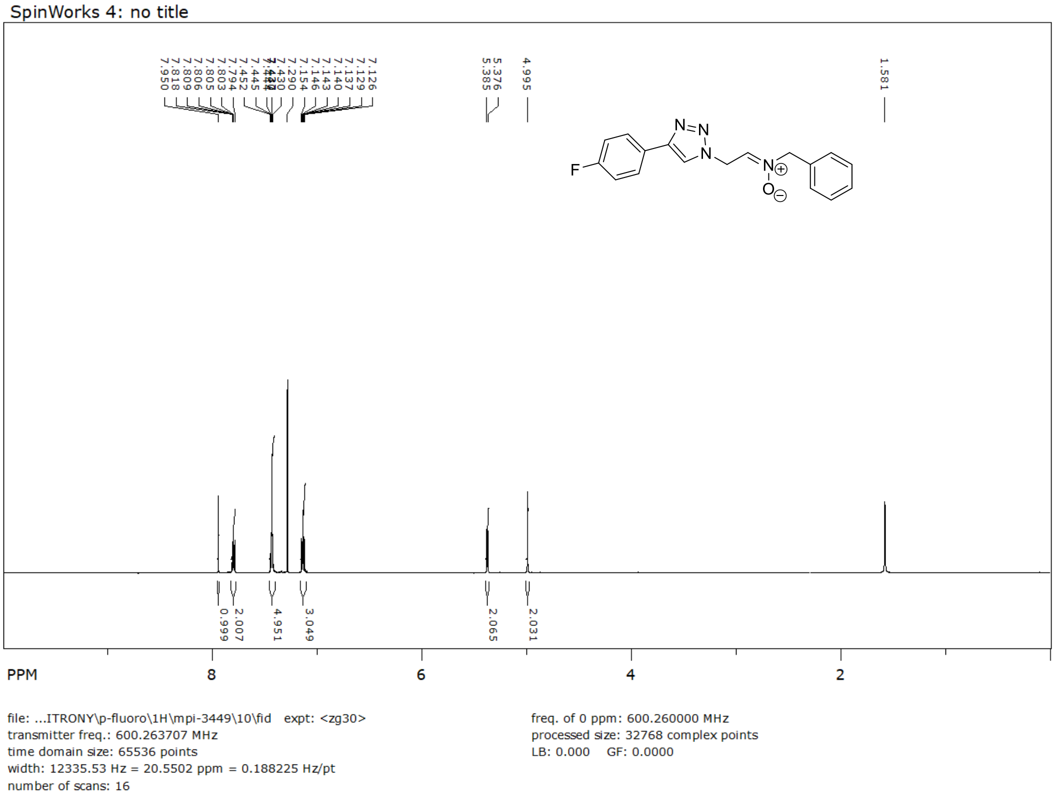

Figure S4: ^{19}F NMR Spectrum for **10b** in $CDCl_3$

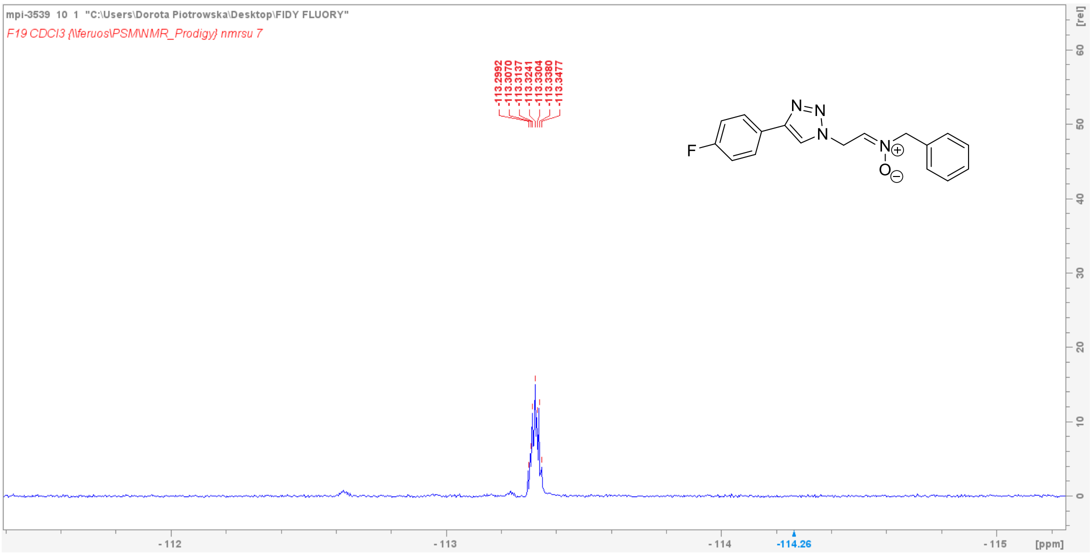

Figure S5: [31]C NMR Spectrum for **10b** in $CDCl_3$

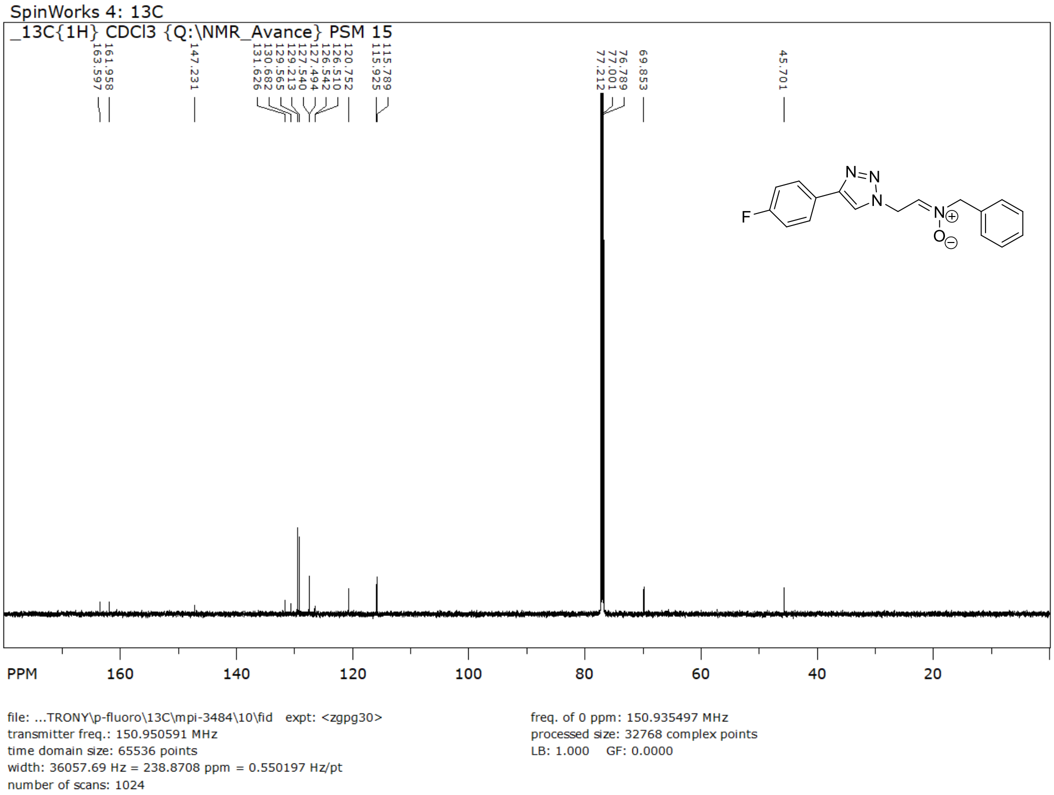

Figure S6: ^{1}H NMR Spectrum for **10c** in $CDCl_3$

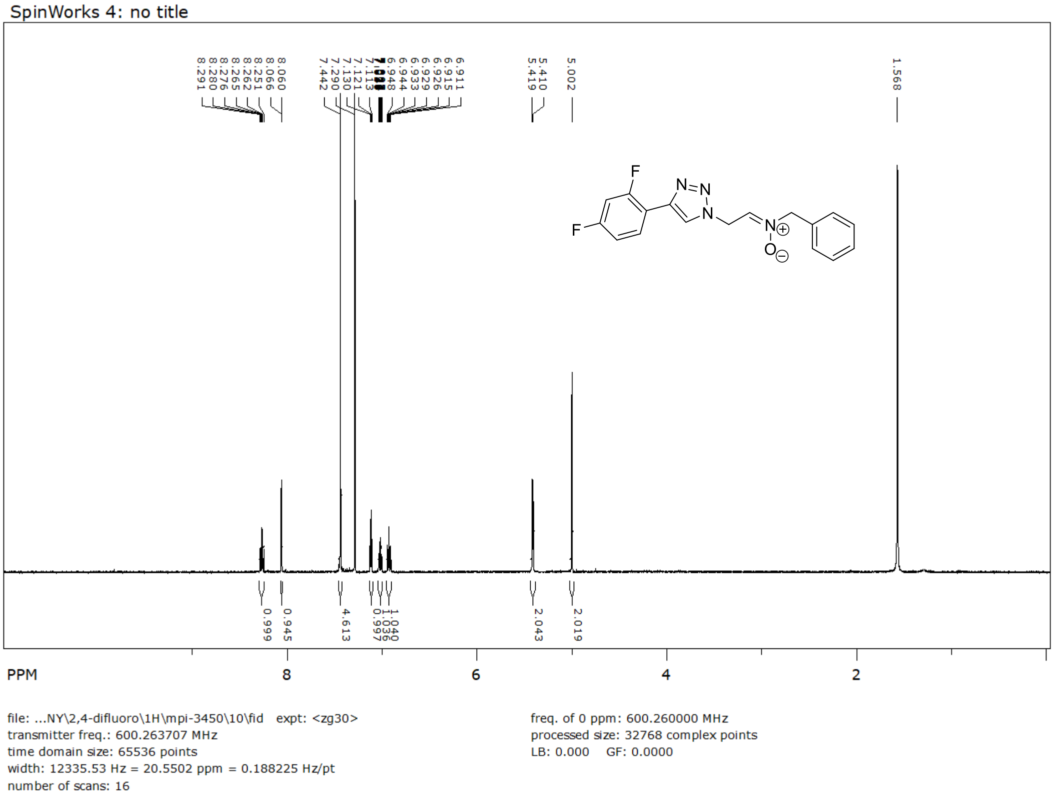

Figure S7: ^{19}F NMR Spectrum for **10c** in $CDCl_3$

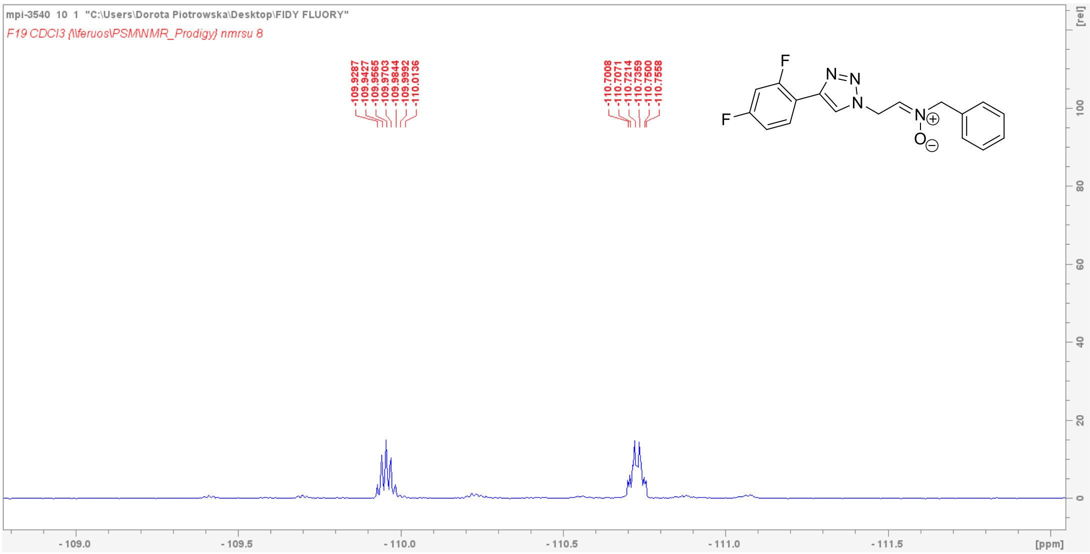

Figure S8: ^{13}C NMR Spectrum for **10c** in $CDCl_3$

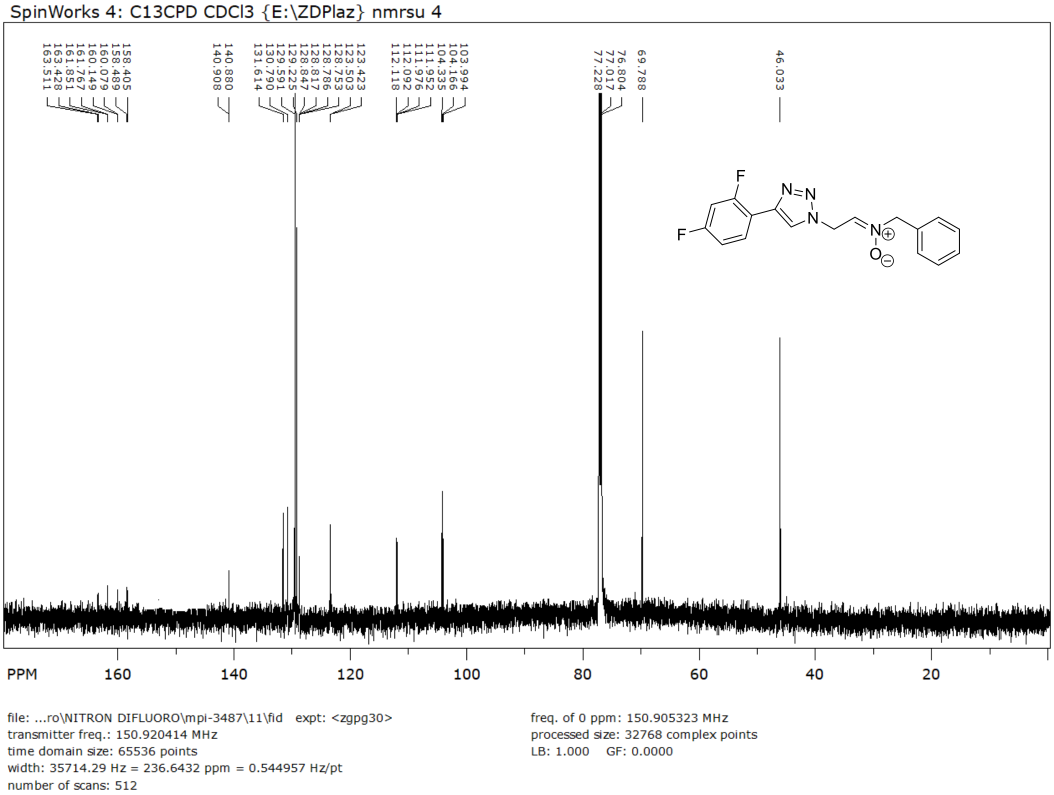

Figure S9: ^{1}H NMR Spectrum for **10d** in $CDCl_3$

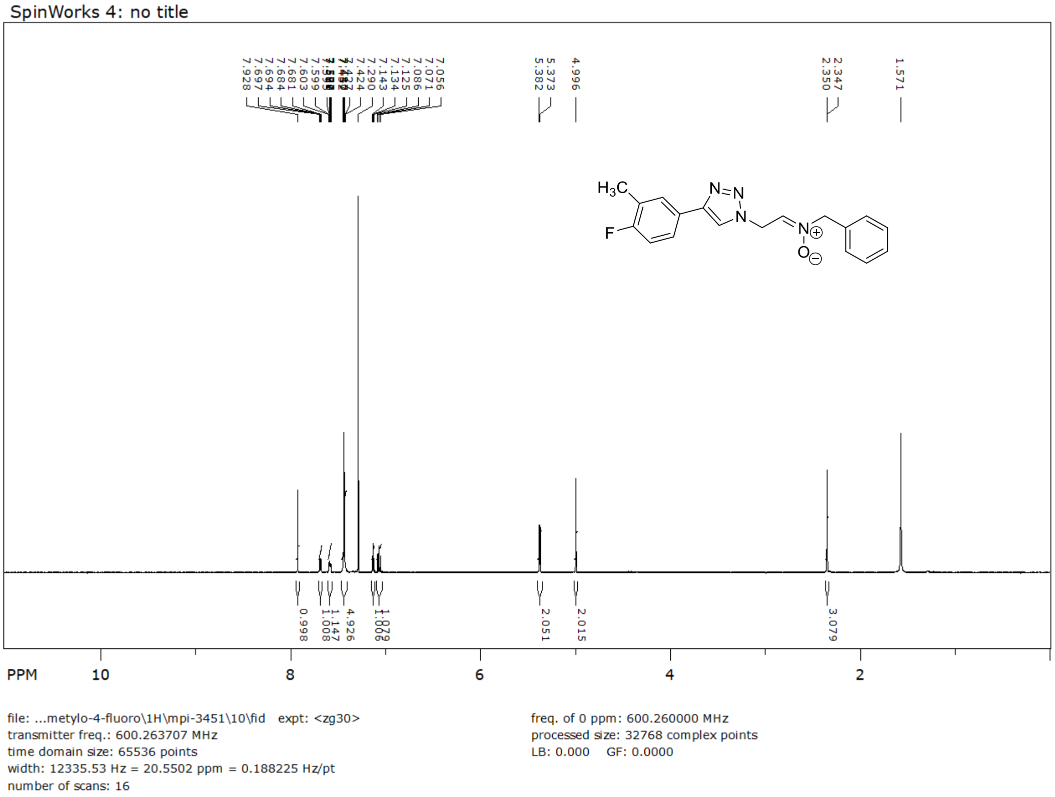

Figure S10: ^{19}F NMR Spectrum for **10d** in $CDCl_3$

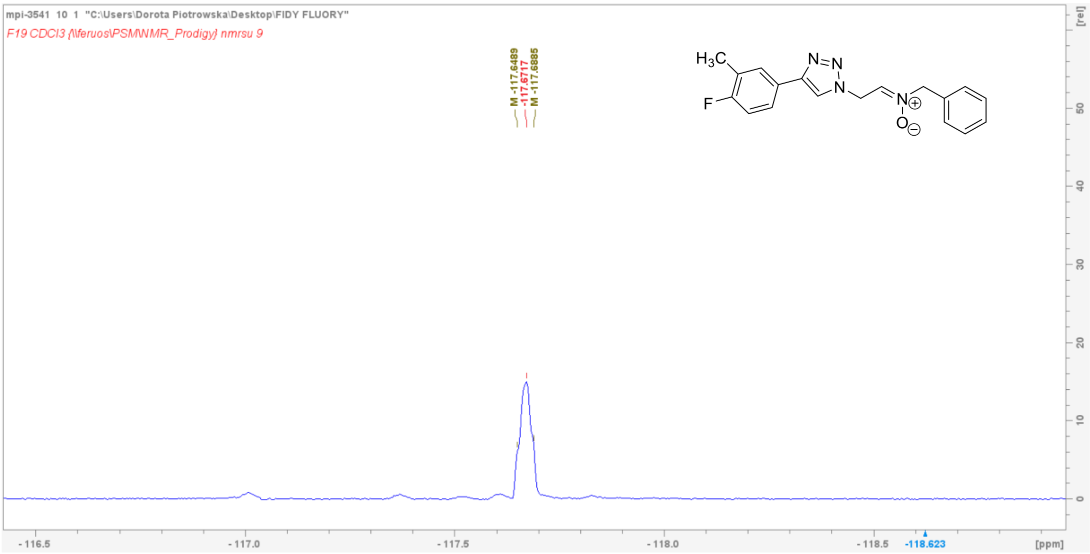

Figure S11: ^{13}C NMR Spectrum for **10d** in $CDCl_3$

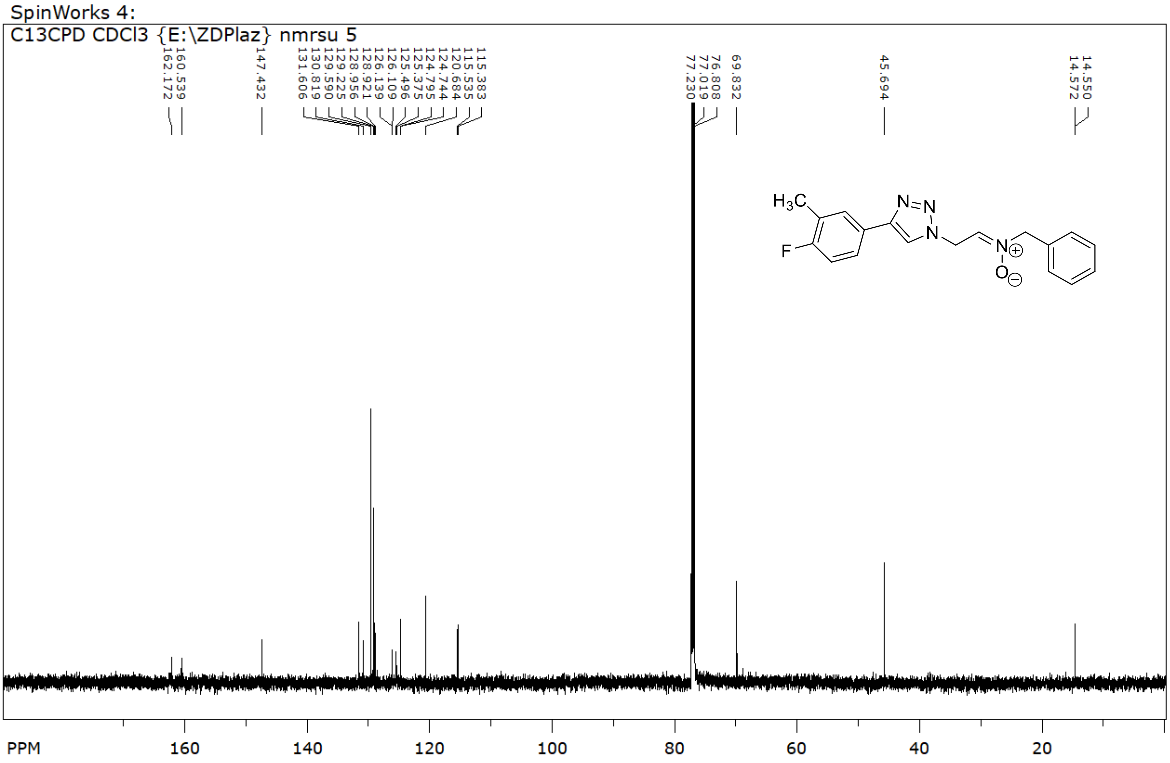

file: ...etylo-4-fluoro\13C\mpi-3488\10\fid expt: <zgpg30>
transmitter freq.: 150.920414 MHz
time domain size: 65536 points
width: 35714.29 Hz = 236.6432 ppm = 0.544957 Hz/pt
number of scans: 128

freq. of 0 ppm: 150.905323 MHz
processed size: 32768 complex points
LB: 1.000 GF: 0.0000
